# Bromide of Potassium in Epilepsy

**Published:** 1872-05

**Authors:** 


BROMIDE OF POTASSIUM IN EPILEPSY.—Dr. J. B. Cox, of French Village, Missouri (*St. Louis Medical and Surgical Journal*), reports eight cases of epilepsy, in which the attacks were cured by the administration of bromide of potassium.

